# Alarm fatigue and sleep quality in medical staff—a Polish-Czech-Slovak study on workplace ergonomics

**DOI:** 10.3389/fpubh.2024.1345396

**Published:** 2024-07-31

**Authors:** Łukasz Rypicz, Izabela Witczak, Mária Šupínová, Hugh Pierre Salehi, Oľga Jarabicová

**Affiliations:** ^1^Department of Population Health, Division of Public Health, Faculty of Health Sciences, Medical University, Wroclaw, Poland; ^2^Faculty of Health Sciences, Catholic University in Ružomberok, Ružomberok, Slovakia; ^3^Department of Biomedical, Industrial, and Human Factors Engineering, Wright State University, Dayton, OH, United States; ^4^Department of Nursing, Faculty of Health Studies, Jan Evangelista Purkyne University in Ústí nad Labem, Ústí nad Labem, Czechia

**Keywords:** alarm fatigue, sleep, healthcare worker, well-being, safety, ergonomics

## Abstract

**Background:**

Alarms are crucial in informing Healthcare Workers (HCWs) about critical patient needs, but unmanaged frequency and noise of alarms can de-sensitize medical staff and compromise patient safety. Alarm fatigue is identified as the major cause of the clinical alarm management problem. It occurs when the medical staff is overwhelmed by the number of clinical alarms.

**Methods:**

The survey was conducted online using Google’s form-making tools from June to July 2023. There were three parts to the survey used in the study: a socio-demographic metric, the Alarm Fatigue Assessment Questionnaire (AFAQ), and The Pittsburgh Sleep Quality Index (PSQI). A significance level of 0.05 was used in the analysis.

**Results:**

The survey included 756 medical professionals from three European countries (Slovakia, the Czech Republic and Poland). The participants in the study were 42 years old on average, and they had 12 years of work experience. 603 out of 756 survey participants had poor sleep quality, 147 had good sleep quality, and 6 did not provide an answer. This study analyzed the alarm fatigue levels of respondents in every country. In the Czech Republic, Poland and Slovakia, a statistically significant association (*p* = 0.039, *p* = 0.001, *p* < 0.001) was found between alarm fatigue and sleep quality in medical staff.

**Conclusion:**

Based on our study, alarm fatigue and sleep quality of HCWs are correlated. Therefore, alarm fatigue and sleep hygiene should be monitored.

## Highlights

- Medical device alarms are a major problem for medical personnel, leading to fatigue.
- Medical personnel’s well-being can be negatively impacted by alarm fatigue.
- The lack of measuring tools makes it hard to monitor alarm fatigue.

## Introduction

Alarms are crucial for letting Healthcare Workers (HCWs) know about critical patient needs, but if alarms are not managed properly, the frequency and noise can de-sensitize medical staff and compromise patient safety (1). Alarm fatigue is a significant issue in healthcare that can be caused by improper alarm management (2, 3). Alarm fatigue is a result of staff being overwhelmed by the number of clinical alarms (2, 4, 5). Alarm fatigue is a safety issue for patients because it can cause alarm desensitization, which can result in delayed or no response from HCWs (4–6). This has led to the Emergency Care Research Institute (ECRI) recognizing alarm fatigue as a significant health technology risk for several years (7).

Determining the number of adverse events caused by alarm fatigue is a challenge, and it is expected that the number is underestimated (8). The reason is that studies report alarm fatigue qualitatively in various ways, including noise level and sensory overload (9–11). Mismanagement and disregarding alarms have resulted in significant deaths, even with current undercounted data. The FDA discovered that alarm mismanagement caused 566 deaths in the US from 2005 to 2010 (4, 12).

Moreover, alarm fatigue has a negative impact on HCWs’ well-being and performance (13, 14).

While providing care, HCWs utilize a variety of medical equipment (15). As a result, they are exposed to multiple alarms, which puts them at risk of alarm fatigue (14). Alarm fatigue increases the risk of burnout, which can lead to mental health issues, such as anxiety and depression (16, 17).

Also, if healthcare workers are exposed to alarms excessively, they may experience hearing irritation, sleep disturbances, and headaches (18). Sleep disturbances are a public health challenge given the importance of sleep for the human body (19). The human body can recover from a day of work during sleep. It is essential to study the quality of sleep and the mechanisms for improving it (20). Due to the high mental workload at work and shift work, HCWs frequently experience sleep problems.

This study aims to demonstrate whether there is a relationship between alarm fatigue and the sleep quality of medical staff. The survey study was conducted in Poland, Slovakia, and the Czech Republic, three countries. These countries were selected based on their cultural and work organization similarities, as well as their geographical proximity.

## Methods

### Study design

After obtaining the Bioethics Committee’s approval, the survey was conducted in three European countries: Poland, Slovakia, and the Czech Republic from June to July 2023. The survey was conducted online using Google’s form-making tools. Each country that participated was given a survey translated into their language. A designated person in each country distributed survey links to practicing medical staff through professional groups such as social media, medical associations, and scientific societies.

After collecting the data, a database was created and analyzed.

This study is exploratory and examines a chosen sample that is not representative of the entire medical staff population. Although it was desirable, the lack of time and resources prevented us from inviting more participants.

The study is intended to focus on medics such as nurses, midwives, doctors, and paramedics who work in departments where medical devices that are alarming are present. These departments are anesthesiology, intensive care unit, cardiac intensive care unit, and recovery room. There were 756 participants in the study, with 455 from Slovakia, 184 from the Czech Republic, and 117 from Poland.

### Research tools

In the study, the survey questionnaire had three parts:

- A socio-demographic metric;
- The Alarm Fatigue Assessment Questionnaire (AFAQ) (21);
- The Pittsburgh Sleep Quality Index (PSQI) (22).

The Alarm Fatigue Assessment Questionnaire (AFAQ) is used to assess alarm fatigue among respondents. The questionnaire score can be anywhere from 20 to 100 points, and a higher number indicates more alarm fatigue. The AFAQ lacks standardized criteria to determine which scores are indicative of high or low alarm fatigue. However, it’s possible to calculate the average score for each question and interpret it. In this study we used the following criteria: 1 means that the feeling of fatigue never occurs, 2 means that it rarely occurs, 3 means that it sometimes occurs, 4 means that it often occurs and 5 means that it always occurs. The AFAQ questionnaire is developed based on Torabizadeh et al. study in Polish language (23).

Following that, the tool was translated into Czech and Slovak. To evaluate the tool’s internal consistency, a Cronbach’s alpha coefficient was calculated. The questionnaire in the Czech version had a Cronbach alpha coefficient of 0.822, and the Slovak version had a Cronbach alpha coefficient of 0.795. A Cronbach’s alpha above 0.7 to 0.8 is acceptable, while 0.8 to 0.9 is good.

The PSQI questionnaire is employed to assess the quality of sleep of participants. Higher scores indicate a lower quality of sleep. In accordance with the key for this scale, the PSQI scores were analyzed, with 0–5 points being indicative of good sleep quality and 6–21 points being indicative of poor sleep quality. In this study we used Polish (24), Czech (25) and Slovak (26) versions of the PSQI.

### Statistical analyses

The distributions of quantitative variables were summarized by using means, standard deviations, medians, and quartiles. Furthermore, the percentage of occurrence was utilized to summarize the distributions of qualitative variables. The chi-squared test was used to compare qualitative variables between groups, with Yates’ correction applied for 2×2 tables. Fisher’s exact test was used to analyze small sample sizes in contingency tables. Quantitative variables between two groups were compared using the Mann–Whitney and Kruskal-Wallis tests for multiple groups. Moreover, the correlation between two quantitative variables was assessed using Spearman’s coefficient of correlation. The significance level for all statistical tests was set to 0.05. The R 4.3.1 program was used for analysis (27).

### Ethics

The study was executed according to the principles of the Declaration of Helsinki and the guidelines of Good Clinical Practice (28). At the top of the survey, there was a section where written consent and study information was provided. The participant identities were not linked to the collected data, and they were free to terminate their participation at any time. The independent Bioethics Committee of Wroclaw Medical University approved the research project (KB 156/2023).

## Results

Females made up the majority of the study participants (94.05% female, 5.95% male). The majority of respondents were nurses (90.21%, *p* = 0.001) and had a mean age of 42 years, with approximately 12 years of work experience. In all three countries, the demographics of the respondents were the same. When taking the survey, the majority of those surveyed, which is 60.32% (*p* < 0.001), were already in a relationship. Nearly all the respondents had equivalent levels of education, with approximately 30% possessing a secondary education that includes a bachelor’s and a master’s degree. Polish participants had the lowest percentage of HCWs with secondary education (*p* < 0.001) compared to Czech and Slovakia. The reason for this is that nurses have not been educated at the secondary level for several decades. Currently, nursing education is offered at the bachelor’s and master’s levels. A total of 72.75 percent (72.75%, *p* = 0.001) work 12-h shifts and have a monthly workload of 160–240 h. The workload is consistent across all countries (*p* = 0.001). Our study shows that 46.15% of Polish healthcare workers work in two locations, which is the highest percentage among survived countries. Table 1 exhibits detailed socio-demographic information.

**Table 1:** Socio-demographic characteristics of the study group.

Parameter		Czechia (*N* = 184)	Poland (*N* = 117)	Slovakia (*N* = 455)	Total (*N* = 756)	*p*
Sex	Female	171 (92.93%)	106 (90.60%)	434 (95.38%)	711 (94.05%)	*p* = 0.114
	Male	13 (7.07%)	11 (9.40%)	21 (4.62%)	45 (5.95%)	
Age [years]	Mean (SD)	41.26 (11.63)	43.26 (11.07)	41.96 (11.37)	41.99 (11.39)	*p* = 0.228
	Median (quartiles)	42 (31.75–50)	46 (34–52)	44 (32–51)	44 (33–51)	
	Range	20–70	22–63	22–65	20–70	
	Missing	0	0	0	0	
Professional experience [years]	Mean (SD)	19.11 (12.39)	20.71 (11.89)	19.21 (12.8)	19.42 (12.56)	*p* = 0.4
	Median (quartiles)	19 (7–30)	22 (9–31)	19 (7–30)	20 (7.75–30)	
	Range	1–50	1–43	0–47	0–50	
	Missing	0	0	0	0	
Marital status	Single	56 (30.43%)	21 (17.95%)	141 (30.99%)	218 (28.84%)	*p* < 0.001 *
	In relationship	105 (57.07%)	95 (81.20%)	256 (56.26%)	456 (60.32%)	
	Divorced/separated	23 (12.50%)	1 (0.85%)	58 (12.75%)	82 (10.85%)	
Profession	Physician	0 (0.00%)	6 (5.13%)	3 (0.66%)	9 (1.19%)	*p* < 0.001 *
	Nurse	175 (95.11%)	93 (79.49%)	414 (90.99%)	682 (90.21%)	
	Midwife	4 (2.17%)	17 (14.53%)	38 (8.35%)	59 (7.80%)	
	Paramedic	5 (2.72%)	1 (0.85%)	0 (0.00%)	6 (0.79%)	
Education	Secondary	100 (54.35%)	5 (4.27%)	135 (29.67%)	240 (31.75%)	*p* < 0.001 *
	Bachelor	53 (28.80%)	33 (28.21%)	167 (36.70%)	253 (33.47%)	
	MSc	31 (16.85%)	79 (67.52%)	153 (33.63%)	263 (34.79%)	
Work system	8-h	56 (30.43%)	24 (20.51%)	101 (22.20%)	181 (23.94%)	*p* = 0.001 *
	12-h	124 (67.39%)	84 (71.79%)	342 (75.16%)	550 (72.75%)	
	24-h	2 (1.09%)	9 (7.69%)	10 (2.20%)	21 (2.78%)	
	Unknown	2 (1.09%)	0 (0.00%)	2 (0.44%)	4 (0.53%)	
Monthly workload	Up to 80 h	7 (3.80%)	2 (1.71%)	2 (0.44%)	11 (1.46%)	*p* < 0.001 *
	80–160 h	49 (26.63%)	46 (39.32%)	105 (23.08%)	200 (26.46%)	
	160–240 h	126 (68.48%)	56 (47.86%)	345 (75.82%)	527 (69.71%)	
	240–320 h	1 (0.54%)	11 (9.40%)	3 (0.66%)	15 (1.98%)	
	Over 320 h	1 (0.54%)	2 (1.71%)	0 (0.00%)	3 (0.40%)	
Working in more than 1 place	Yes	57 (30.98%)	54 (46.15%)	118 (25.93%)	229 (30.29%)	*p* < 0.001 *
	No	127 (69.02%)	63 (53.85%)	337 (74.07%)	527 (69.71%)	
Do you like your work?	Yes	177 (96.20%)	110 (94.02%)	429 (94.29%)	716 (94.71%)	*p* = 0.682
	No	7 (3.80%)	7 (5.98%)	22 (4.84%)	36 (4.76%)	
	Unknown	0 (0.00%)	0 (0.00%)	4 (0.88%)	4 (0.53%)	
Residence	Rural area	60 (32.61%)	30 (25.64%)	168 (36.92%)	258 (34.13%)	*p* < 0.001 *
	City up to 50 th. inhab	42 (22.83%)	14 (11.97%)	116 (25.49%)	172 (22.75%)	
	City 50–150 th. inhab	42 (22.83%)	23 (19.66%)	91 (20.00%)	156 (20.63%)	
	City 150–500 th. inhab	30 (16.30%)	19 (16.24%)	54 (11.87%)	103 (13.62%)	
	City over 500 th. inhab	10 (5.43%)	31 (26.50%)	26 (5.71%)	67 (8.86%)	

*p*, Qualitative variables: chi-squared or Fisher’s exact test. Quantitative variables: Kruskal-Wallis test. ^*^Statistically significant (*p* < 0.05).

The survey found that 603 out of 756 survey participants (79.76%) had low quality sleep (6–21 PSQI points), while 147 out of 756 participants had good sleep quality (0–5 PSQI points) and 6 participants did not answer this question. Slovakia and Poland had significantly higher sleep problems than the Czech Republic (*p* < 0.05), and the PSQI scores for Poland and Slovakia were relatively similar (Table 2). All three countries scored above 5 points, which suggests that healthcare workers in all three countries are experiencing poor sleep quality.

**Table 2:** Overall sleep quality score for each country.

Country	*N*	PSQI [points]							*p*
		Mean	SD	Median	Min	Max	Q1	Q3	
Czechia (CZ)	182	7,59	3,29	7	1	19	5	10	*p* < 0.001 *
Poland (PL)	117	8,78	3,53	8	1	19	6	11	SK, PL > CZ
Slovakia (SK)	451	8,80	3,37	9	1	19	6	11	

*p*, Kruskal-Wallis test + post-hoc analysis (Dunn test); SD, standard deviation; Q1, lower quartile; Q3, upper quartile.^*^Statistically significant (*p* < 0.05).

Alarm fatigue levels were calculated for participants in every country in this study. The statistical differences between countries are significant (*p* < 0.05). Alarm fatigue caused by medical devices was significantly higher in Slovakia and Poland than in the Czech Republic (Table 3). Alarm fatigue and sleep problems are positively correlated (Table 4). All three countries had statistically significant positive correlations, with the correlation coefficients being 0.171 (*p* = 0.039) in the Czech Republic, 0.303 (*p* = 0.001) in Poland, and 0.228 (*p* < 0.001) in Slovakia. The importance of this result for further exploration is that it supports the idea that medical devices that emit audible alarms have an impact on the sleep quality of medical staff. Furthermore, the association between socio-demographic data and alarm fatigue was investigated. Alarm fatigue has been found to have a statistically significant relationship (*p* < 0.05) with age and length of service. Poland, Czech Republic, and Slovakia show a correlation coefficient of −0.28; −0.378; −0.156, which indicates that more years of practice result in a reduction in alarm fatigue experience. The prevalence of alarm fatigue in older adult HCWs is lower than it is in younger individuals, as assessed by Spearman’s correlation coefficients for Poland, Czech Republic, and Slovakia: −0.274, −0.362, −0.169. Alarm fatigue was found to be significantly greater in those working 12-h shifts than in those working 8-h shifts only in the Czech study group (*p* < 0.05). There was no statistical significance observed for other demographic variables and alarm fatigue.

**Table 3:** Overall alarm fatigue score for each country.

Country	*N*	AFAQ [points]							*p*
		Mean	SD	Median	Min	Max	Q1	Q3	
Czechia (CZ)	148	37,17	9,16	36	21	64	30	42	*p* < 0.001*
Poland (PL)	114	40,85	10,31	39	23	69	33	47	SK, PL > CZ
Slovakia (SK)	379	40,53	8,95	40	21	73	34	47	

*p*, Kruskal-Wallis test + post-hoc analysis (Dunn test); SD, standard deviation; Q1, lower quartile; Q3, upper quartile.^*^Statistically significant (*p* < 0.05).

**Table 4:** Relationship between sleep quality and fatigue alarms by country.

Country	Variables	Spearman’s correlation coefficient	*p*
Czechia	AFAQ & PSQI	0.171	*p* = 0.039 *
Poland	AFAQ & PSQI	0.303	*p* = 0.001 *
Slovakia	AFAQ & PSQI	0.228	*p* < 0.001 *

^*^Statistically significant (*p* < 0.05).

## Discussion

Healthcare workers’ well-being and patient safety depend on their sleep quality. This article explores the relationship between alarm fatigue and the quality of sleep of healthcare workers. Sleep hygiene can lead to an increase in job satisfaction for healthcare staff and, ultimately, a healthier and more resilient healthcare system. Alarm fatigue is a result of HCWs being overexposed to alarms, which can lead to indifference and potentially death or permanent injuries for patients (15, 29). The impact of medical device alarms on patient and staff safety has become a growing concern in healthcare facilities.

According to Bourjet al., Alarm fatigue and its consequences are affecting all medical personnel (30).

The effects of alarm fatigue on the well-being of healthcare workers have only been investigated in a few studies at present. Our study shows that alarm fatigue is a common occurrence among medical staff because they are exposed to medical alarms for a long time at work. Alarm fatigue issues are present in all participating countries in our study, which is concerning. Alarm fatigue was more intense in Poland and Slovakia than in the Czech Republic, among the three participating countries in this study.

Healthcare workers are increasingly mentioning that alarm fatigue has a negative impact on sleep quality when discussing its impact on their well-being. Curry et al. showed that sleep disturbance occurs due to alarm fatigue (31). The study discovered that alarm fatigue has a negative impact on the quality of sleep of medical staff. Similarly, Kaylor et al. conducted a study to investigate alarm fatigue and sleep quality in caregivers of children with diabetes who continuously monitor glucose levels. The quality of sleep for caregivers is negatively impacted by alarm fatigue, as evidenced by the results (32).

The reduction of alarm fatigue can be achieved with effective management of medical device alarms (33). It is crucial to prioritize this issue as prolonged exposure to this psychosocial risk factor can lead to decreased quality of life, depression, job burnout, or cardiovascular problems among HCWs, as well as patient safety risks (34–37). There is no question that this is an area of research that needs further examination due to its direct impact on the safety of patients and healthcare workers and education is crucial to solving this issue.

## Conclusion

Healthcare systems worldwide are facing a challenge due to the shortage of healthcare workers. The current shortage can be worsened by the presence of psychosocial stressors in hospitals and clinics, causing absenteeism and resignations. Medical staff shortages can result in negative outcomes and pose a potential risk to both patient safety and the well-being of medical staff.

Medical alarms have the potential to cause significant stress for medical staff, which can further exacerbate the situation. Excessive exposure to alarms can lead to fatigue and decreased sleep quality. Long-term fatigue caused by sleep disturbances can pose significant health and patient safety risks. Just like other psychosocial risk factors, the exposure to medical alarms requires effective monitoring and management in hospitals and clinics.

Based on our study, alarm fatigue and sleep quality of HCWs are correlated and the Alarm Fatigue Assessment Questionnaire (AFAQ) and The Pittsburgh Sleep Quality Index (PSQI) can be employed to measure alarm fatigue and evaluate sleep hygiene. Medical staff and patients can be more satisfied and safer by continuing monitoring for alarm fatigue.

## Study limitations

It’s worth noting that despite the invitation to all health professionals (doctors, paramedics, and nurses/midwives) to participate in the study, nurses received the highest response rate. Therefore, the study group cannot represent all health professions due to the low response rate of other professions when compared to nurses. Given the significant proportion of respondents being women, it is important to consider gender as a limitation. Continuing research in this stream should consider aspects such as non-sound alerts, setting and adjusting limits on alarms, the impact of OSA and obesity on sleep quality, and the prevalence of hearing problems in staff versus responding to alarms.

## Data availability statement

The raw data supporting the conclusions of this article will be made available by the authors, without undue reservation.

## Author contributions

ŁR: Conceptualization, Data curation, Formal analysis, Funding acquisition, Investigation, Methodology, Project administration, Resources, Software, Supervision, Validation, Visualization, Writing – original draft, Writing – review & editing. IW: Formal analysis, Writing – original draft. MŠ: Resources, Software, Validation, Writing – original draft. HS: Writing – original draft, Writing – review & editing. OJ: Methodology, Resources, Writing – original draft.
