# Correction: Alarm fatigue and sleep quality in medical staff—A Polish-Czech-Slovak study on workplace ergonomics

**DOI:** 10.3389/fpubh.2025.1729764

**Published:** 2025-10-31

**Authors:** Łukasz Rypicz, Izabela Witczak, Mária Šupínová, Hugh Pierre Salehi, Ol'ga Jarabicová

**Affiliations:** ^1^Department of Population Health, Division of Public Health, Faculty of Health Sciences, Medical University, Wroclaw, Poland; ^2^Faculty of Health Sciences, Catholic University in Ružomberok, Ružomberok, Slovakia; ^3^Department of Biomedical, Industrial, and Human Factors Engineering, Wright State University, Dayton, OH, United States; ^4^Department of Nursing, Faculty of Health Studies, Jan Evangelista Purkyne University in Ústí nad Labem, Ústí nad Labem, Czechia

**Keywords:** alarm fatigue, sleep, healthcare worker, well-being, safety, ergonomics

The funder Wroclaw Medical University, project number SUBK.E260.23.002 was erroneously omitted. The correct **Funding** statement reads

The author(s) declare financial support was received for the research and/or publication of this article. This study received internal resources funding from: REZD.2 507.24.003. This study was carried out as part of a project implemented at the Wroclaw Medical University, project number SUBK.E260.23.002.

The original version of this article has been updated.

